# Hereditary Neuroses in Children.—VII

**Published:** 1897-07-03

**Authors:** 


### Modern Sociology.

### HEREDITARY NEUROSES IN CHILDREN.—VII.

Having in previous papers reviewed the varied phenomena of hereditary neurosis in children, and endeavoured to follow, as far as practicable, their etiological factors, it only remains for us, in conclusion, to gather up some sociological lessons suggested by our study of the subject.

The laws of inheritance are complex, and heredity may be described as direct, reversional, initial, or prenatal. We have seen that, fortunately for the race, morbid variations are kept in check by Nature's tendency to revert to the normal type. When, however, her good intentions are frustrated by the ignorance or obstinacy of man, and continued morbid inheritance is entailed by injudicious marriages, her Nemesis is the extinction of the family which persists in propagating the unfit.

Without entering upon a discussion of the doctrines advanced by Weismann, we may accept Ribot's definition of heredity as "that biological law by which all beings endowed with life tend to repeat themselves in their descendants." If direct, the repetition of physical and mental qualities is from parents to child; if reversional, from grandparents (or more remote ancestor) to grandchild, the parental generation escaping the impress; if collateral, the characters seen in the uncle, aunt, or cousin, rather than those of the parents, are stamped upon the child. In the collateral, as well as the reversional variety, there is, no doubt, a common cause at work, often designated atavism, by virtue of which the attributes of a more or less remote ancestor, latent in the parental stock though evidenced in collateral branches, is brought into prominence. The individual child's character is, moreover, influenced by the predominance of particular qualities in the parents at the time of conception (constituting "initial heredity"), and by the environments and health of the mother during gestation ("prenatal heredity").

If these principles regulate the general inheritance of physical and mental characteristics, they apply in a special manner to the morbid inheritance of nervous disorders. One cannot always trace in the parents the neurosis which is manifested in the child; but if we go back a stage further, and scrutinise the grandparental and previous generations, we shall probably discover some heritable nervous affection, the existence of which may also be evidenced in the collateral line of uncles, aunts, and cousins. In the case of the three children referred to in a former paper* constituting an insane family, the only indications of neurotic heredity was that the mother's father's sister had been insane, though, of course, evidence on such a subject is not easily obtained. Indeed, anything like a complete family history can hardly be expected with ordinary work-a-day folks; and we have to look to royal and noble houses for illustrations of transmission and transformation of neurosis through many successive generations. One such genealogy has been worked out by Dr. Ireland in his book entitled "The Blot upon the Brain," in the case of the Royal Family of Spain from 1449 to 1700. This shows how a neurotic taint, followed through eight or nine generations, manifests itself, with variable intensity, under the form of epilepsy, hypochondria, mania, melancholia, and imbecility, sometimes skipping a generation, and finally leading to the extinction of the race. Consanguineous marriages are frequent in the legitimate line, and it is worthy of note that the illegitimate descendants of the Spanish kings seem to have escaped the neuropathic troubles of those born in wedlock, thus showing the salutary influence of "new blood."

* The Hospital, May 29th, p. 141.

Of consanguineous marriages it may be remarked that their danger is the risk of intensification in the offspring of any family taint derived by the parents from a common ancestry. Given a perfectly healthy stock, such unions may be unobjectionable, and they were even commended in the early ages of the world as a means of keeping up a select royal caste in the ancient dynasties of Persia and of Egypt. But in these latter days few families can show a faultless physiological pedigree, and the marriage of near kin is, as a rule, to be deprecated. The existence of any neurosis in the line of common ancestry should be regarded as an absolute bar to such a union.

What, then, are the practical lessons to be learnt from these observations on heredity? First and foremost the need of caution in matrimonial selection as viewed from the physiological standpoint. Age, good looks, social position, and wealth are objects of interest for intending suitors; but how often does the consideration of family history come into the question? Yet with a bad heredity on both sides it may be a case not merely of "marry in haste, and repent at your leisure"; but the repentance may extend to generations unborn. If we have not yet arrived at sufficiently exact information to formulate legal conditions of matrimonial fitness, we know enough to justify the prohibition to propagate their kind in the case of confirmed lunatics, constitutional dipsomaniacs, and habitual criminals. Not to speak of human happiness, such restrictions may be based on regard for social economy. In the "study made by Dugdale of the Jukes' family in New York it was shown that from a single feeble-minded woman descended many generations of paupers and criminals, while the worst of vices characterised the majority of her descendants. Records were made of 709 persons who were descendants of this woman. 52 per cent. of all the women in the number were prostitutes. In the 709 persons were 76 criminals."* And, that such evils are not confined to America, the following history, culled from an English newspaper, will show: "On September 13th, 1895, there died at Chester a John Ogden, who had made 130 appearances before the city justices, 86 being for drunkenness and 44 for assaults. Ogden's father appeared before the bench 35 times, a sister 67 times, and another sister 29 times. The father, son, and two sisters were charged altogether 247 times. For expenses of prosecution, prison and poor-law maintenance, the Ogden family have cost Chester £2,000." In the State of Connecticut it is a felony for any epileptic, imbecile, or weak-minded woman to marry while the woman is under the age of forty-five years; and in another state an even more drastic scheme (for unsexing the unfit) has been brought before the legislature. In this old country we think more slowly, but there is evidence that public sentiment is gradually being educated to see the necessity of restricting the reproduction of morbid taints; and especially those of which we have been treating under the title of hereditary neuroses.

* American *Journal of Psycho-Æsthenics*, Dec. 1896, p. 62.

